# Searching for a Signal: Self-Reported Kratom Dose-Effect Relationships Among a Sample of US Adults With Regular Kratom Use Histories

**DOI:** 10.3389/fphar.2022.765917

**Published:** 2022-03-01

**Authors:** Kirsten E. Smith, Jeffrey M. Rogers, Kelly E. Dunn, Oliver Grundmann, Christopher R. McCurdy, Destiny Schriefer, David H. Epstein

**Affiliations:** ^1^ Real-World Assessment, Prediction, and Treatment Unit, National Institute on Drug Abuse Intramural Research Program, Baltimore, MD, United States; ^2^ Department of Psychiatry and Behavioral Sciences, Johns Hopkins University School of Medicine, Baltimore, MD, United States; ^3^ Department of Medicinal Chemistry, College of Pharmacy, University of Florida, Gainesville, FL, United States

**Keywords:** kratom, *Mitragyna speciosa*, dosing, use patterns, kratom withdrawal, kratom effects

## Abstract

There is limited understanding regarding kratom use among US adults. Although motivations for use are increasingly understood, typical kratom doses, threshold of (low and high) doses for perceived effectiveness, and effects produced during cessation are not well documented. We aimed to extend prior survey work by recruiting adults with current and past kratom exposure. Our goal was to better understand kratom dosing, changes in routines, and perception of effects, including time to onset, duration, and variability of beneficial and adverse outcomes from use and cessation. Among respondents who reported experiencing acute kratom effects, we also sought to determine if effects were perceived as helpful or unhelpful in meeting daily obligations. Finally, we attempted to detect any signal of a relationship between the amount of kratom consumed weekly and weeks of regular use with ratings of beneficial effects from use and ratings of adverse effects from cessation. We conducted an online survey between April-May 2021 by re-recruiting participants from a separate study who reported lifetime kratom use. A total of 129 evaluable surveys were collected. Most (59.7%) had used kratom >100 times and reported currently or having previously used kratom >4 times per week (62 weeks on average). Under half (41.9%) reported that they considered themselves to be a current “regular kratom user.” A majority (79.8%) reported experiencing acute effects from their typical kratom dose and that onset of effects began in minutes but dissipated within hours. Over a quarter reported that they had increased their kratom dose since use initiation, whereas 18.6% had decreased. Greater severity of unwanted effects from ≥1 day of kratom cessation was predicted by more weeks of regular kratom use (*β =* 6.74, *p* = 0.02). Acute kratom effects were largely reported as compatible with, and sometimes helpful in, meeting daily obligations. In the absence of human laboratory studies, survey methods must be refined to more precisely assess dose-effect relationships. These can help inform the development of controlled observational and experimental studies needed to advance the public health understanding of kratom product use.

## 1 Introduction

### 1.1 History

The plant indigenous to Southeast Asia, *Mitragyna speciosa*, commonly referred to among Westerners as “kratom,” has been used in the United States and other regions outside of Asia since at least 2004 (Burkill, 1935; Boyer et al., 2007; Boyer et al., 2008). In Asia, particularly Malaysia and Thailand, kratom preparations have been used for medicinal, cultural, energy-enhancing, and recreational purposes and to decrease heroin and amphetamine misuse without significant adverse effects documented to date (Singh et al., 2016; Singh et al., 2019a; Singh et al., 2020a; Leong Bin Abdullah et al., 2020; Ramanathan and McCurdy, 2020; Saref et al., 2020; Singh et al., 2021). Although there is speculation that kratom was introduced into the US contemporaneous to the Vietnam War, it is unclear when kratom use in the US began in earnest (Legislative Analysis and Public Policy Association, 2019). As far back as 1988, researchers began to note the plant’s therapeutic potential as a replacement for or supplement to methadone treatment among people with opioid use disorder (OUD; Jansen and Prast, 1988) and by 2016 it was apparent that kratom was being used by persons with and without clinical disorders, including persons with opioid and other substance use disorder (SUD) histories (Boyer et al., 2008; Swogger et al., 2015; Grundmann, 2017; Smith and Lawson, 2017).

### 1.2 Reasons for Use

Motivations for using kratom have become the topic of numerous case reports and surveys (Griffiths et al., 2018; Agapoff and Kilaru, 2019; Aldyab et al., 2019; Coe et al., 2019; Stanciu et al., 2019; Bowe and Kerr, 2020; Covvey et al., 2020; Garcia-Romeu et al., 2020; Schmuhl et al., 2020; Smith et al., 2021a; Weiss and Douglas, 2021; Grundmann et al., 2022a). Case reports, including those of kratom-associated fatalities, are insightful but provide limited detail and generalizability beyond the clinical presentation(s) described in the report (Olsen et al., 2019; Post et al., 2019). Most do not specify motivations for kratom use and focus largely on adverse effects, given the medical context. Larger epidemiological level surveys have been conducted with samples in the US; these studies provide more definitive understandings of kratom use motivations. However, these are also somewhat limited in their use of convenience samples of current, regular kratom-using adults who self-select into kratom-specific survey participation. Regular and current use can make such respondents a good source of information, but could conceivably contribute to response bias, in that they may have favorable attitudes about kratom use compared to infrequent or remitted users. Put differently, people who have quit using kratom likely did so for a reason (which might include having found the effects unremarkable) and therefore may be less inclined to participate in a kratom survey. Conversely, some people may be regular current users due to an inability to stop.

Nevertheless, these larger surveys have been able to elucidate many broad motivations for why persons may be using kratom, such as the self-treatment for chronic pain, fatigue, psychiatric, or SUD symptoms or to improve energy, mood, and enhance recreation generally (Grundmann, 2017; Swogger and Walsh, 2018; Coe et al., 2019; Bath et al., 2020; Garcia-Romeu et al., 2020). These reports corroborate findings from Southeast Asia (Singh et al., 2016; Singh et al., 2017; Singh et al., 2019a; Singh et al., 2020b; Müller et al., 2020). Another commonly cited reason for kratom use both in the US and Asia includes reducing, substituting, or stopping licit or illicit substances, the most common being opioids, though kratom use to abstain from alcohol or amphetamine is also reported (Saingam et al., 2013; Singh et al., 2020b; Vicknasingam et al., 2020; Singh et al., 2021). These reports converge with analyses of social-media posts and online content (Smith et al., 2021b; Smith et al., 2021c; Prevete et al., 2021; Grundmann et al., 2022b). Collectively data suggest that kratom use motivations, practices, and consequences are continuing to evolve in the US, and that frequent updates are required.

### 1.3 Pharmacology of Kratom

Four of kratom’s over 40 known bioactive alkaloids, mitragynine (MG), 7-hydroxymitragynine (7-HG), corynoxine, and speciociliatine, appear to act at μ-opioid receptors. The two most heavily studied, MG and 7-HG, seemingly act as partial opioid receptor agonists, though non-opioid actions are also observed with these and other alkaloids (Kruegel and Grundmann, 2018; Fowble and Musah, 2019; Kruegel et al., 2019; Obeng et al., 2019; King et al., 2020; Todd et al., 2020; Berthold et al., 2021; Chear et al., 2021; Kamble et al., 2021). MG and 7-HG have been found to produce a range of mostly dose-dependent acute and chronic effects (both adverse and potentially therapeutic) that are consistent with μ-opioid receptor activity in nonhuman animals, including: discriminability as opioids (with partial generalization to psychostimulants); self-administration; conditioned place preference; attenuation of opioid self-administration and opioid withdrawal; and analgesic, antinociceptive, and anxiolytic effects (Hazim et al., 2014; Harun et al., 2015; Yusoff et al., 2017; Yue et al., 2018; Hemby et al., 2019; Hiranita et al., 2019; Hassan et al., 2020; Kamble et al., 2021; Obeng et al., 2021; Suhaimi et al., 2021). The complexity of the kratom botanical and variability of its alkaloid composition is influenced by the environmental conditions in which it grows and by harvesting or post-harvest handling practices (Zhang et al., 2012; Griffin et al., 2016; Lydecker et al., 2016; Prozialeck et al., 2020).

### 1.4 Understanding How Kratom Dosing Corresponds to Effects

Kratom-based survey studies have rarely provided sufficient detail to determine what constitutes a “typical” or “regular” dose. Without a specific unit of measurement, it is not possible to determine the threshold at which kratom may produce specific effects. The need for specificity is supported by studies that have found associations between use patterns and outcomes (Ahmad and Aziz, 2012; Saingam et al., 2013; Singh et al., 2014; Singh et al., 2019b; Phillip, 2019; Müller et al., 2020). For instance, Grundmann (2017) found that most participants (57.5%) experienced no negative (withdrawal-like) effects if kratom was not taken at 12-, 24-, and 48-h increments, and, among those who did experience negative effects when not using kratom, those effects were rarely characterized as severe. Coe et al. (2019) reported high variability in the prevalence of adverse effects, ranging from 0.8% (hallucinations) to 76.8% (stomach problems), though the lack of dosing information limits interpretation of these results. Garcia-Romeu et al. (2020) did collect data on dosing from persons regularly using kratom in the US and found the typical dose range was <1 g (8.6%) to >7 g (8.9%), with most respondents reporting that they consumed 1–3 g (49.0%) or 4–6 g (33.4%) per consumption. This finding was contextualized by number of doses per week, with most respondents reporting they consumed kratom daily, primarily as a prepared beverage (37.0%) or ingesting it as raw powder (43.6%) or capsule (18.9%). Most in that sample reported mild or no adverse effects, however effects as a function of dose were not examined. One notable example is Grundmann (2017) who reported odds ratios for both beneficial and adverse effects for amount/dose and doses/week finding that most beneficial effects were observed in doses of 1–3 and 3–5 g if taken 2–3 times per day; in contrast, most adverse effects required higher doses of >8 g and higher frequency of dosing between 4–5 times per day of daily use.

These data contrast and converge with some effects described in social-media posts, wherein some individuals recounted moderate to severe effects with prolonged use at higher doses (tolerance or withdrawal symptoms), and a wide-ranging beneficial effects at specified and unspecified doses (Smith et al., 2021a; Smith et al., 2021b). But the social-media data, too, lack context in that they do not quantify dosages or durations systematically. Complicating matters further is that kratom products have changed considerably since 2017, with new products available and with greater diversity and (largely unknown) variability across vendors and products in quality and alkaloid content.

### 1.5 Aims

We aimed to expand upon the data provided by prior studies that focused primarily on establishing the prevalence and motivations for kratom use by adding information about kratom dose conventions. This study aimed to depart from prior studies that enrolled persons with current kratom use, to include persons who had lifetime exposure to kratom but may not be using it currently. In this way, we hoped to reduce the potential for positive bias among respondents and ensure that some respondents had ceased their use; the goal of this approach was to collect a balanced perspective on the relative benefits and consequences of kratom exposure. Data were analyzed to support more precise and contextualized understanding of kratom dosing routines, changes in routines, and perception of effects—including time to onset, duration, and perceived effectiveness in terms of reasons for use. We consider the majority of findings here primarily descriptive. Among respondents who reported ever having periods of regular use, we also wanted to try to evaluate whether dosage (the amount of kratom consumed per week, and weeks of regular use) corresponded to the number of beneficial and/or aversive effects reported. Although we suspected there would be a directional association, with lower weekly doses being more likely associated with beneficial effects and higher doses more likely to be associated with adverse effects, we did not test this as a priori hypotheses, given the early state of human kratom (self-report) research and the uncertainty surrounding the statistical power we would be able to achieve when we recontacted our kratom-using respondents from a prior survey (see below). The pragmatic goal of this recontact survey was to collect descriptive data that would inform a follow-up study using ecological momentary assessment (EMA). Specifically, we needed to learn more about the time frames along which we should make multiple daily momentary random and dose-dependent assessments, as well as the types of questions we should ask (e.g., whether to assume that kratom is typically used for its acutely perceptible effects and not for chronic effects like a maintenance medication).

## 2 Materials and Methods

### 2.1 Study Procedures

Amazon Mechanical Turk (mTurk), an online platform for crowdsourcing research participation and other online tasks requiring human interaction, was used here for recruitment, screening, and compensation (Chandler and Shapiro, 2016; Sheehan, 2018). mTurk is regularly used for obtaining national convenience samples in behavioral and substance use research and for ensuring the capacity to obtain valid data (Peer et al., 2014; Shank, 2016; Miller et al., 2017; Mortensen and Hughes, 2018; Sheehan, 2018; Strickland and Stoops, 2018; Strickland and Stoops, 2020). “Workers” are persons who register in mTurk who are then enabled to volunteer to participate in research by choosing to accept “human intelligence tasks” (HITs) that are presented to workers who meet broad study inclusion criteria or who may be eligible and are subsequently screened for eligibility. Workers can also decline to accept, or participate in, a screening HIT. No personally identifiable information was collected in our surveys (except for IP addresses, which were deleted after verification as US addresses). The studies involving human participants were reviewed and approved by the National Institutes of Health Institutional Review Board. They were given exempt status, meaning that written informed consent was not obtained from participants, just assent.

The present kratom survey study was a follow-up to a prior mTurk-based online survey study on substance use and social conditions (unrelated to kratom). That study enrolled people who met the following inclusion criteria: >18 years; US residents; a registered mTurk worker account with >100 completed HITS, and past-six-month use (≥1 day of use during the 6 months prior to screening) for one of the following two categories: 1) alcohol only (nicotine and caffeine permitted, but not illicit drug use); 2) opioids or psychostimulants. Opioid use was defined as licit opioids (prescription opioid analgesics, prescribed methadone, and/or prescribed buprenorphine), illicit opioids (heroin, fentanyl, nonmedical/diverted prescription opioids, and/or nonmedical/diverted methadone or buprenorphine), and kratom. Psychostimulant use was defined as illicit psychostimulants [powder or crack cocaine, synthetic cathinones, “street” methamphetamine, 3,4-methylenedioxymethamphetamine (MDMA), or diverted psychostimulant medications]. Participants were admitted into that study if they endorsed using alcohol only or any of these opioid or stimulant substances, independent of whether other drugs were also endorsed.

Data collection for the larger survey study hosted on Qualtrics occurred between September 2020 and March 2021. A total of 13,608 screening questionnaires were completed. Of these, 3,414 (25.1%) met inclusion criteria; of these, 2,864 completed the full survey. To ensure data quality, four data validity checks were programmed into the screening questionnaire and 26 were programed into the full survey. Failing 1 validity check during screening or >3 during the full survey, or exceeding the 4-h survey completion time, resulted in automized unenrollment. Of the 2,864 completers, 249 cases were removed for one of the following reasons: unrealistically short completion time, discrepant screener and full survey items (e.g., drug use history, demographics), IP address outside of the US, IP address of indeterminate location, VPN or proxy IP address that made it impossible to validate respondents’ US location. Thus, the final sample included 2,615 valid surveys.

### 2.2 Kratom Recontact Survey

Persons who endorsed lifetime use of kratom on that larger, unrelated study were recontacted and asked to provide additional information related to kratom. This strategy resulted in a convenience sample of US adults with kratom use history who had not (initially) self-selected based on their kratom use. Data from respondents who reported lifetime kratom use are the basis of the analyses presented here. A total of 289 respondents (of the 2,615) from the larger survey study endorsed lifetime kratom use and passed all data quality checks. Re-recruitment of and data collection from these 289 respondents for this kratom survey occurred during a 1-month period (April 15–May 15, 2021). We sent reminders at two time points. Those who were successfully recontacted and chose to participate were compensated $7.25.

### 2.3 Kratom Survey Instrument and Study Measures

We developed a kratom survey instrument based on the current literature. Given the rapid changes in kratom products available to US consumers and the ever-evolving landscape of kratom use in the US, we included items from prior surveys, but also new or refined items, based on our prior research, clinical experience, and questions needed to inform new projects. Here we focus on findings specific to relationships between dosage and effect (or some indicator of those relationships). Additional findings from the kratom recontact survey are reported elsewhere. Our survey instrument may be made available upon request.

#### 2.3.1 Sample Characteristics

Sample characteristics included age, sex/gender, race/ethnicity, education (high school graduate, college graduate), past-year employment (part- or full-time), and past-year household income (below vs. above US Federal poverty line, for entire household).

#### 2.3.2 Kratom-Use History

Kratom-use history was assessed first at the time of screening into the larger study survey, by asking respondents if they had used kratom during their lifetime or past year (timeframes were exclusive). On the kratom recontact survey, we collected additional details, such as age of kratom use initiation. For comparison, we also asked respondents to report the ages at which they had first used nicotine (e.g., cigarettes), alcohol, and cannabis, as these are commonly used, both in the US and among persons with kratom use histories. Lifetime kratom use disorder (KUD) was assessed using a modified DSM-5 checklist for substance use disorder (SUD) that was modified for kratom use (e.g., “I spent a great deal of time on activities necessary to get kratom, use kratom, or recovery from kratom’s effects”; “I experienced cravings, strong desires, or urges for kratom”). These items were presented so as to evaluate whether respondents had ever qualified for kratom use disorder, regardless of current use [see Smith et al. (2022) for full KUD findings].

#### 2.3.3 Kratom Dose Units

Typical dose was measured by having respondents select the formulation by which they most frequently consumed kratom: capsule, gram, spoonful, tablespoon, cups of tea, etc. These units were selected to reflect the ways in which kratom consumption has been previously reported. Respondents were asked to then indicate the amount in numerical units per dose (e.g., 2 g) and the typical number of doses per day for their preferred method of administration. Respondents then reported on the length of time that they had been using this typical daily amount in weeks, months, or years (coded in weeks here).

#### 2.3.4 Kratom Dosing Routines

Regularity of kratom use was assessed by asking respondents to answer (yes/no) to the items: “Have you used kratom more than 100 times in your lifetime?” “Was there ever a period of time during which you used kratom at least 4 times per week?” and “What was the longest period during which you used kratom at least 4 times per week?” to which they could respond with numerical value in weeks, months, years (coded in weeks here). Respondents were also asked whether they considered themselves to currently be “a regular kratom user” (yes/no).

Daily patterns of kratom dosing were assessed, in part, using modified questions from the Fagerström Test for Nicotine Dependence (FTND) (Heatherton et al., 1991) by asking respondents: “How soon after you wake do you use your first dose of kratom?” to which they could respond: “Within 5 min,” “6–30 min,” “31–60 min,” or “after 60 min” Respondents could then respond to the items “Do you take kratom more during the first hours after waking than during the rest of the day?” (yes/no) and “Which kratom dose would you hate most to give up?” (“The first one in the morning” vs. “All other times of day”).

Changes in dosing routines were assessed by asking people to respond to items from the FTND found to be strongly associated with current nicotine dependence (Baker et al., 2007): “Since you first began using kratom (during times of active use), how often did you change your dosing routine?” to which they could respond on a 5-point Likert scale with an additional response option (1 = very often to 5 = never; 6 = It depends on other circumstances). Changes in frequency of kratom dosing since initiation were assessed by having respondents endorse one of the following options: “increased,” “unchanged,” “decreased,” “never took kratom regularly,” and “have quit entirely.”

#### 2.3.5 Kratom Perceived Effects as a Function of Dose

We asked all respondents: “When you take kratom, do you feel an effect pretty much every time? These could be energy-boosting effects, like those of a cup of coffee, or intoxicating effects, like those of an alcoholic beverage—or anything else you feel from each dose.” The three response options were: “Yes, I feel an effect every time (or almost every time) I take kratom,” “No, I never (or rarely) feel an effect when I take kratom,” and “Neither of those is quite true for me (elaborate if you’d like).”

For those who reported feeling effects from each dose, we asked: “If you do feel the effects with each dose of kratom, are they primarily helpful in letting you go about your daily obligations?” The six response options were: “Yes, the effects are compatible with my daily obligations and help me achieve them,” “Yes, the effects are compatible with my daily obligations, though not especially helpful for them,” “No, the effects are not compatible with my daily obligations,” “No, the effects are not compatible with my daily obligations and they sometimes undermine my ability to meet my daily obligations,” “I don’t take kratom enough to know,” and “None of those are quite true for me (elaborate if you’d like).” For those who did not report feeling effects from each dose, we asked: “If you don’t usually feel effects with each dose of kratom, which of these options best describes why you use it?” The three response options were: “I don’t want effects with each dose: I use kratom just for its long-term effects, the way some people use antidepressants or other medications,” “I feel withdrawal symptoms if I stop using it,” and “Neither of those is quite true for me (elaborate if you’d like).”

Respondents were also asked to report how long it typically took them to begin feeling the effects from their usual dose of kratom (in seconds, minutes, or hours, or “I don’t know”). An identical question was asked regarding how long it typically took them to stop feeling the effects of their usual dose in minutes or hours; respondents could also select the option “I’m unsure because I would take more kratom before the effects would wear off.” We then assessed respondents’ perceived dose effectiveness *via* the number of units (e.g., grams, capsules). Specifically, respondents reported a “too low” kratom dose that they found to be ineffective (defined for them as not producing their desired results); a lower-threshold dose (the lowest dose that was effective in producing their desired results); an upper-threshold dose (the highest dose that was effective as intended without being too high), and a “too high” dose (a dose for which the resultant effect that was “a bit too much” or produced effects that were not wanted or intended).

#### 2.3.6 Perceived Beneficial and Adverse Effects

Motivations for use that represented beneficial or desired effects were assessed by asking respondents to select the most important factors that influenced or motivated current or past kratom use. They were then presented with 41 use-motivation categories (e.g., chronic pain management; relieve opioid withdrawal symptoms, relieve alcohol withdrawal symptoms; self-treat anxiety; for recreation). Respondents could select all applicable use indications. Respondents were then asked to rate effects on these outcomes selected using visual analogue scale (VAS) sliders (0–100), with 0 reflecting “not at all effective” and 100 reflecting “extremely effective” (see Supplementary Table S1 for the complete list of beneficial use motivations endorsed and subsequently rated). Analyses were conducted using pooled VAS, for which the number of respondents who endorsed any effect and the mean and standard deviation for all summed VAS ratings of kratom effects were determined.

Motivations for use that represented an unwanted effect or negative reinforcement (and specifically included avoidance of withdrawal) were assessed in a similar manner. Respondents were asked “What unpleasant or unwanted side effects have you experienced when you have stopped taking kratom at least for a period of 1 day or longer? These could be considered withdrawal or withdrawal-like effects.” They were then presented with a list of 23 adverse effects (e.g., depression or sadness; irritability; body aches; restless legs; stomach upset) which, when selected, were rated using a VAS slider wherein 0 reflected intensity of withdrawal-like effects as “almost nothing” and 100 reflected “severe or unbearable discomfort.” (see Supplementary Table S1). This question, and list of response items, was based upon our prior research examining kratom withdrawal symptoms (Smith et al., 2022). Outcomes were evaluated again as independent scales and then pooled across 21 effects (those that were endorsed). Finally, respondents were provided with an open-text response option to the item, “Please describe the adverse effects you have personally experienced as a result of using kratom.” All text responses were categorized for summary presentation in order to characterize adverse effects associated with kratom use.

### 2.4 Data Analysis

We generated means and proportions for the entire sample for all descriptive items. Demographic data include self-report from the larger survey. All other data were obtained from persons who completed the kratom recontact survey (n = 129). Our overarching goal was to provide a description of kratom doses, changes in doses, effects, and compatibility of acute kratom effects with daily life, as perceived by persons who experience acute effects and have regularly used kratom.

In addition to these characterizations we wanted to examine, to the extent possible with the sample size, the effect of dose on kratom-related outcomes that included standardized ratings of beneficial effects that respondents attributed to kratom use and standardized ratings of unwanted or adverse effects attributed to discontinuation of kratom use (for ≥1 day).

To accommodate differences in consumption methods (e.g., capsules, grams, spoonfuls, tablespoons, or cups), we standardized reported amounts by calculating within-unit z-scores for each consumption method. Preliminary analyses included Pearson and Spearman tests for correlation or t-test (findings from preliminary analyses are provided in Supplementary Material). Due to the sample size, statistical significance in univariate or bivariate analyses was not what we relied on to determine variable inclusion for the final models, in part because we wanted to see how the variables interacted when all were included in the model, including covariates. Independent variables pertaining to use and dosing amounts were selected for examination as we believed they had the potential to demonstrate relation to reported kratom effects. Specifically, we assessed the relative role of dose by fitting two linear regression models that examined dose and other person-level predictors of experiencing beneficial effects of kratom or unwanted/negative effects after missing ≥1 day of use. Main predictors included in the two models were: self-reported regular kratom dose amount (kratom consumed per week), weeks of regular use, changes in use (decreased, increased, or quit versus unchanged dose), and endorsement of frequently using kratom more within the first hour after waking (yes/no). Potentially confounding demographic factors were included as covariates. For the first model, the dependent variable was pooled VAS ratings for beneficial effects attributed to as motivators for use; for the second model the dependent variable was pooled VAS ratings for unwanted effects of cessation (≥1 day). Primary outcomes of interest were to determine a relationship between each respective dependent variable and: 1) the amount of kratom consumed per week and, 2) number of weeks of regular kratom use (unstandardized). Model parameters were generated using ordinary least squares regression. Continuous explanatory variables were mean-centered and factor variables were included in the model as dummy codes. As such, model intercepts can be interpreted as the mean VAS score for people of average levels of each continuous variable and the reference level of each factor variable. Regression beta (*B*) values displayed in Table 5 are unstandardized and represent the change in VAS scores as an explanatory variable increases by one unit, holding all other explanatory variables constant. Significance tests for individual regression betas were conducted using single degree of freedom tests represented by t-values (t) and *p*-values (p). Model error distributions were evaluated for normality and identity, and to ensure that potential assumption violations did not negatively impact model estimates; we conducted a sensitivity check with Box-Cox transformed response variables and determined that model predictions were robust to response variable transformations. Collinearity was assessed *via* variance inflation factors (VIF).

## 3 Results

Of the 289 eligible mTurk workers who reported lifetime kratom use in our larger survey study, 6 no longer had active mTurk worker IDs and were unable to participate in our kratom recontact survey, making 283 people eligible. From those 283, we received (n = 134, 47.4%) complete responses during the 1-month data collection period. Five cases were removed due to inability to verify IP addresses, providing us with a final sample of 129. A majority of responses (59.7%) were submitted by respondents who had reported past-month kratom use on the larger survey.

### 3.1 Sample Characteristics and Kratom-Use History

Table 1 displays sample demographics and kratom-use history. Persons using kratom in this sample were on average 34.8 ± 8.4 years old (±indicates standard deviation), female (51.9%), white (71.9%), high school (40.3%) or college educated (59.7%), and employed at least part-time (68.2%). Just under a quarter reported an annual household income below the US poverty line.

**TABLE 1:** Means and proportions for sample demographic characteristics, kratom use history, typical dosing routines, and changes in kratom dosing. Total sample (n = 129).

	N reporting	%	Mean	SD
Age	129		34.84	(±8.4)
Female	67	51.90		
White	102	71.90		
High School graduate	52	40.30		
College graduate	77	59.70		
Past-year employment (at least part-time)	88	68.20		
Past-year household income below Federal poverty line	28	21.70		
Age of kratom use initiation (range 16–60)	129		29.9	(±8.8)
Age of combustible nicotine use initiation (not electronic)	120		15.9	(±4.5)
Age of alcohol use initiation	128		15	(±3.3)
Age of cannabis use initiation	122		16.8	(±5.4)
Qualified for lifetime kratom use disorder	40	31.00		
Has used kratom >100 times during lifetime	77	59.70		
Has ever (regularly) used kratom >4 times per week	104	80.60		
Weeks spent using kratom >4 times per week	104		61.9	(±104.3)
Currently considers themselves a regular kratom user	54	41.90		
Kratom doses per day	104		2.68	(±1.73)
Weeks spent using typical dosing routine	129		65	(±112.9)
Typical regular kratom dose				
Capsules	47		5.38	(±4.76)
Grams	37		4.57	(±3.61)
Spoonfuls	25		2.52	(±2.71)
Tablespoons	11		2.09	(±1.04)
Cups of Teas	8		1.62	(±1.06)
Shots	1		1.00	(±0.0)
First dose after waking				
<5 min	6	4.70		
6–30 min	30	23.30		
31–60 min	21	16.30		
>60 min	72	55.80		
Uses kratom more during the first waking hour than other times	53	41.10		
The kratom dose that you would most hate to give up?				
First one of the morning	70	54.30		
All other times of day	59	45.70		
During periods of regular use, frequency of change in dosing routine				
Very often	10	7.80		
Often	12	9.30		
Occasionally	42	32.60		
Not often	37	8.70		
Never	12	9.30		
Depends on the circumstances	16	12.40		
Since kratom use initiation, the frequency of dosing has				
Increased	34	26.30		
Unchanged	29	22.50		
Decreased	24	18.60		
Never took kratom regularly enough to note a change	12	9.30		
I have quit entirely	27	20.90		
Other	3	2.30		

Respondents reported first using kratom at 29.9 ± 8.8 years of age, on average. A majority had used combustible nicotine, alcohol, or cannabis, with initiation ages that were on average far younger than those for kratom (15.9, 15.0, and 16.8 years, respectively). Most (59.7%) had used kratom >100 times during their lifetime and reported currently or having previously used kratom >4 times per week, for an average of 61.9 ± 104.3 weeks (80.6%). Just under half (41.9%) considered themselves current “regular” kratom users. Nearly one-third met diagnostic criteria for lifetime KUD.

### 3.2 Typical Kratom Dosing Routines and Changes

**FIGURE 1:**
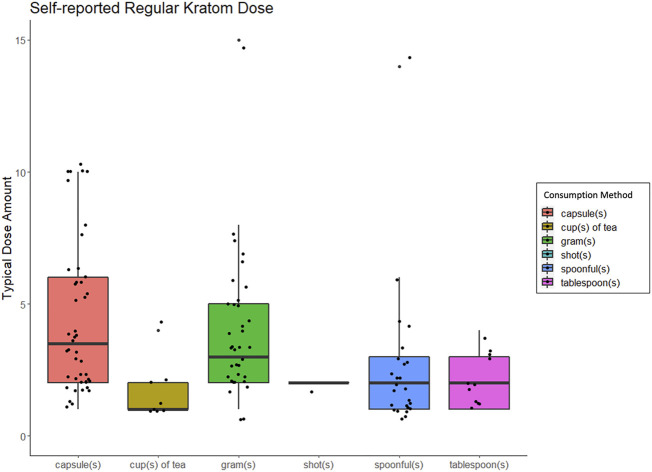
Box and whisker plots displaing people's self-reported "regular" or "typical" kratom dose, broken down by method of kratom consumption.

As shown in Table 1, respondents most frequently reported consuming kratom *via* capsules (n = 47), grams (n = 37), spoonfuls (n = 25), tablespoons (n = 11), and then cups of tea (n = 8). The average amount of kratom used per unit was 5.4 ± 4.8 capsules, 4.6 ± 3.6 g, 2.5 ± 2.7 spoonfuls, 2.1 ± 1.0 tablespoons, or 1.6 ± 1.1 cups of tea. See Figure 1 for typical kratom dosing units. On days that people used kratom, they reported dosing 2.6 ± 2.4 times and that this typical dosing routine had been stable for 65.0 ± 112.9 weeks.

A slim majority of respondents (55.8%) reported that they did not typically take kratom until they had been awake for at least an hour; 28.0% typically consumed kratom within the first 30 min after waking. Additionally, a sizeable minority (41.1%) reported consuming more kratom during the first waking hour than at other times during the day and 54.3% reported preferring their first daily kratom dose of the morning to those consumed during other times of day. When asked how often they changed their dosing routine during periods of regular use, most reported changing only “occasionally” (32.6%) or “not often” (8.7%). Some said instead that their dosing routine changes were dependent on circumstances (12.4%). Only 7.8% reported changing their dosing routine “very often.” Since initiating use respondents described their dose amounts as having increased (26.3%), or remained unchanged (22.5%), though nearly as many said it had decreased (18.6%); 20.9% had quit.

### 3.3 Kratom Perceived Effects

Table 2 displays descriptive findings pertaining to kratom perceived effects. Most (79.8%) indicated that they experienced an acute subjective effect with each dose they consumed; only 7.0% did not. Of the 13% who indicated “neither of those is quite true for me”; their free-text responses to this probe noted fluctuations in their own tolerance, effects that varied for uncertain reasons (n = 5), effects that varied by dose (n = 3), or effects that varied by product (n = 3).

**TABLE 2:** Means and proportions for kratom perceived acute effects, compatibility of effects with daily obligations, and effectiveness and ineffectiveness by dose. Total sample (n = 129).

	N	%	
Acute Effects			
Felt an effect every time (or almost every time) kratom was dosed	103	79.80%	
Never or rarely felt an effect when kratom was dosed	9	7.00%	
Neither of these is quite true for me	17	13.20%	
“Typically, how long would it take for you to *begin* to feel the effects of your typical dose of kratom?”			
Seconds	0	0.00%	
Minutes	107	82.90%	
Hours	15	11.60%	
I don’t know	7	5.40%	
“Typically, how long would it take for you to *stop* feeling the effects of your usual dose of kratom?”			
Minutes	2	1.60%	
Hours	118	91.50%	
I’m unsure because I would take more kratom before the effects would wear off	9	7.00%	
Among those who reported feeling the effects from each dose (n = 103)			
The kratom effects are compatible with *and* help me meet my daily obligations	56	54.40%	
The kratom effects are compatible with, but do *not* help me meet my daily obligations	30	29.10%	
The kratom effects are *not* compatible with my daily obligations	4	3.90%	
No, the effects are not compatible with my daily obligations, *and* they sometimes undermine my ability to meet daily obligations	3	2.90%	
I don’t use kratom enough to know if effects are compatible or helpful daily	9	8.70%	
None of those are quite true for me	1	1.00%	
	**N**	**Mean**	**SD**
“Too low” dose (at which kratom was *ineffective)*			
Capsules	50	3.96	(±4.95)
Grams	45	2.64	(±2.44)
Spoonfuls	19	1.37	(±0.96)
Tablespoons	5	2.2	(±2.17)
Cups of Teas	7	1.57	(±0.98)
*Lower-threshold* dose at which kratom was *effective*			
Capsules	45	4.13	(±3.31)
Grams	43	3.19	(±2.25)
Spoonfuls	24	2.33	(±2.2)
Tablespoons	6	2	(±0.89)
Cups of Teas	10	1.3	(±0.67)
*Upper-threshold dose (highest* dose at which kratom was *effective as intended)*			
Capsules	43	5.88	(±4.02)
Grams	40	6.85	(±4.58)
Spoonfuls	23	2.87	(±1.58)
Tablespoons	10	2.5	(±1.58)
Cups of Teas	8	2.25	(±1.16)
*“Too high” d*ose (at which the effect “a bit too much”, or produced results that were not wanted, intended, or effective)			
Capsules	40	7.25	(±4.24)
Grams	37	8.68	(±4.38)
Spoonfuls	27	3.93	(±1.66)
Tablespoons	7	3.57	(±1.72)
Cups of Teas	9	3.44	(±2.01)
Pooled “positive” (beneficial or therapeutic) kratom effects (VAS 0–100)	104	72.84	(±16.73)
Pooled “negative” (adverse or unwanted) kratom effects (VAS 0–100)	104	52.98	(±24.12)

Among the 103 respondents who reported experiencing acute effects from kratom, 54.4% reported that those effects were compatible with their daily life and helped them to meet daily obligations; 29.1% reported that kratom effects were compatible with, but did not necessarily help them meet, daily obligations. Only 3.9% reported that kratom effects were not compatible with their daily obligations, and 2.9% reported that kratom effects sometimes outright undermined their ability to meet daily obligations. A small number (8.7%) reported that they did not take enough kratom to know, and one chose the response option “None of these is quite true for me,” explaining in free text that in her few experiences with kratom, she had sought a caffeine-like effect but had found the effect more like “two glasses of wine.”

Twenty-five respondents completed the item asking why they took kratom in the absence of acute effects. Of those, 6 (4.7% of the full sample) indicated they were seeking chronic benefits rather than acute effects; 3 (2.3% of the full sample) reported using kratom to avoid withdrawal, and 16 (12.4% of the full sample) felt “Neither of those is quite true for me.” A follow-up free-text option revealed some were continuing to try for acute effects despite tolerance (or, as some suggested, “bad product,” n = 4), using kratom to prevent withdrawal from other opioids (n = 2), using kratom for help with energy or pain relief (n = 2), and that cessation caused rebound pain and continued dosing therefore seemed beneficial (n = 1).

No respondent reported feeling kratom’s subjective effects within seconds, whereas 82.9% reported typically beginning to feel kratom’s effects within minutes; with lower endorsement of hours (11.6%). Nearly all (91.5%) reported that they typically stopped feeling kratom’s effects within hours, with 2 respondents reporting feeling that the effects stopped within minutes; 7.0% reported that they were unsure of the duration of effects due to the fact that they consumed more kratom prior to the effects of the prior dose fully dissipating.

**FIGURE 2:**
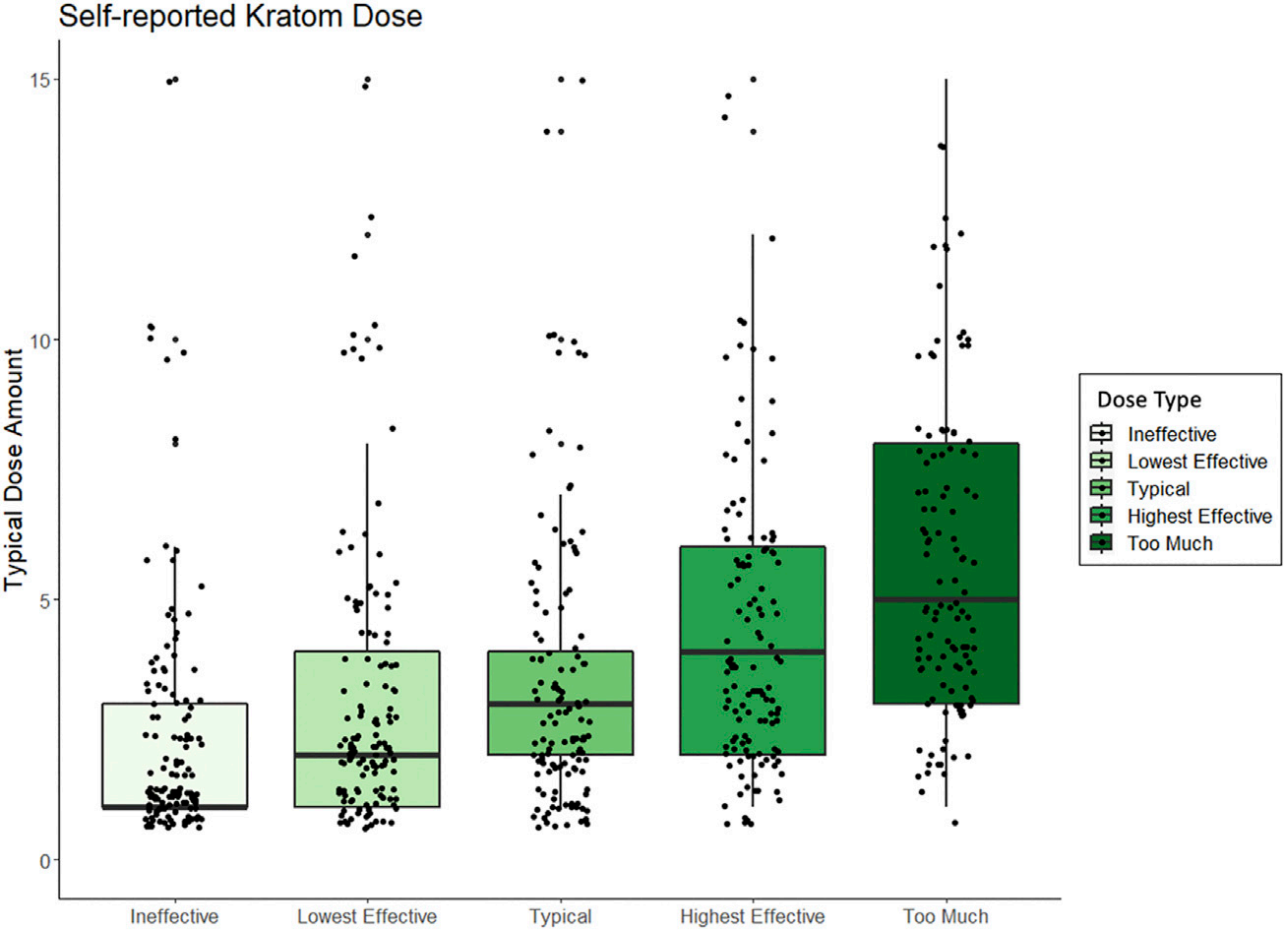
Box and whisker plots displaying peoples self-reported kratom doses, standardized across method of kratom consumption, and which range from amounts that people perceived to be “ineffective” to what dose they perceived to be “too much.”

As shown in Table 2, the mean dose of kratom that respondents felt was “too low” (e.g., unable to elicit desired effects) was 3.96 capsules (n = 50), 2.64 g (n = 45), 1.37 spoonfuls (n = 19), 2.2 tablespoons (n = 5), or 1.57 cups of tea (n = 7). The mean lower-threshold dose of kratom (the lowest effective dose) was reported as 4.13 capsules (n = 45), 3.19 g (n = 43), 2.33 spoonfuls (n = 24), 2.00 tablespoons (n = 6), or 1.3 cups of tea (n = 10). The mean upper-threshold doses (effective as intended without unwanted effects) was reported as 5.88 capsules (n = 43), 6.85 g (n = 40), 2.87 spoonfuls (n = 23), 2.5 tablespoons (n = 10), or 2.25 cups of tea (n = 8). The mean “too high” dose (perceived to be “a bit too much”) was reported as 7.25 capsules (n = 40), 8.68 g (n = 37), 3.39 spoonfuls (n = 27), 3.57 tablespoons (n = 7), or 3.44 cups of tea (n = 9). Figure 2 shows these data by reported dose amount and type. See Supplementary Material for figures displaying self-reported effects for grams, capsules, spoonful’s, tablespoons, and cups of tea, respectively.

Column 1, Supplementary Table S1 in supplementary material lists the 41 items that respondents could endorse and rate (for perceived effectiveness) as the most important factors that motivated their past or current kratom use. These included: “self-treating anxiety symptoms,” “reliving withdrawal from nonprescribed opioids or heroin,” and to “boost energy, stamina and/or endurance (for work, exercise).” Ratings of the effectiveness of these beneficial kratom effects were used to calculate the average perceived effectiveness of kratom for all use indications. The average perceived effectiveness of kratom across all reported use indications, was 72.8/100 (±16.7.) Column 2, Supplementary Table S1 in supplementary material lists the items (e.g., nausea, hot flashes, running nose) that respondents could endorse and rate as being experienced when they stopped taking kratom ≥1 day. For these unwanted effects of cessation (≥1 day), the average pooled severity rating was 53.0/100 (±24.1).

### 3.4 Perceived Adverse Effects From Kratom Reported *via* Open Text Responses

Table 3 lists unwanted effects that respondents reported *via* open-ended questions that they had perceived resulting from their kratom use. Those included gastrointestinal upset (nausea, vomiting, constipation, cramping), low mood (dysphoria, difficulty concentrating, anxiety) and a variety of somatic symptoms (increased urination, dehydration, dry mouth, rash). Table 4 provides direct quotes from some open-ended responses to contextualize findings (full text data available upon request).

**TABLE 3:** Adverse or unwanted side effects reported by participants as being directly caused by kratom use *via* open text response quantified in raw number and percent frequency (n = 129).

	N	%
Nausea	36	27.9
None	16	12.4
Vomiting	14	10.9
Constipation	14	10.9
Headaches	13	10.1
Increased feelings of anxiety/nervousness	12	9.3
Withdrawal symptoms	8	6.2
GI upset	7	5.4
Bad taste	7	5.4
Tiredness	7	5.4
Dizziness	6	4.7
Bad mood	5	3.9
Addictive/developed dependence	5	3.9
Dehydration	4	3.1
Inconsistent (wobbly) eye movement	4	3.1
Trouble sleeping	4	3.1
Stomach cramping	4	3.1
Increased feelings of depression	3	2.3
Jittery/restless	3	2.3
Tolerance	2	1.6
Irritated skin/rash/itch	2	1.6
Dry mouth	2	1.6
Increased heart rate	2	1.6
Increased perspiration	2	1.6
Cramps/body aches	1	0.8
Weight gain	1	0.8
Trouble focusing	1	0.8
Fluctuating mood	1	0.8
Speech issues	1	0.8
Restless leg syndrome	1	0.8
Craving for tobacco	1	0.8
Increased urination	1	0.8
Decreased motivation	1	0.8
Light headedness	1	0.8
Decreased appetite	1	0.8

**TABLE 4:** Direct quotes from participants who provided open text responses about adverse or unwanted kratom side effects.

“I felt nauseous one time while experimenting with dosages in the first 2 weeks of regular use (I think I took around 15 g which I never do anymore, but I could probably handle it now).” “Sometimes it hits different and don’t produce the same effect and can be frustrating but that could be a number of factors.” “Nausea, the awful taste, gagging from having to put so much of the powder form in a tea, the smell. Light headaches but manageable.” “Headaches happen sometimes especially if I dose too early, nausea on occasion, constipation.” “With too much kratom on an empty stomach I’ve gotten increased heart rate and nervousness.” “The times after I was over withdrawal from heroin and clean i used kratom to get over meth. It didn’t work at all. Made me high like opioids then ill. And every time after I have ever used it any color I just get sick. Like mentally and physically.” “It often makes me very tired after it wears off or if I take repeated doses over consecutive days. It dries my mouth out a lot.” “Sometimes I would take my regular dose and I would get so freaking sick it’s not even funny. The world would spin. My stomach would crap and feel like I needed to throw up so badly. Horrible headache to the point my eyes were sore.” “Too much kratom would make me feel agitated and anxious. I also wouldn’t be hungry.” “I’ve puked before if my dose was too big or my stomach to empty; its rather rare at this point more frequent when I was new to it and figuring out dosage.”	“Only thing was constipation when I first started “using” kratom, but it is long gone. I can get restless leg syndrome if I don’t have any before bed, but it’s not terrible.” “After the high wears off, I actually get unmotivated. I don’t like how addictive it is for a plant.” “The only adverse effects I have experienced from kratom has been constipation at times. If I don’t drink enough water or eat enough fiber I end up needing to take a laxative. The only other adverse effect is that after a year of being on it every day, if I don’t take it I feel pretty bad but it doesn’t compare to heroin or methadone withdrawal. Also sometimes if I take too much by accident or intentionally I get the wobbles. The wobbles are what kratom users refer to as nausea and dizziness from taking too much. When that happens you need to lay down.” “Never OD’d. I don’t think you can. If you take too much you get shaky in your eyes that’s called the wobbles. Also if you already eat fiber, this stuff will clog you up it’s so fibrous. On the other hand if you are like me and eat protein bars and air for all her meals, kratom also saved my digestive system because it firms up your stools!” “Have experienced withdrawal on a few occasions after periods of extended use; am aware that I will definitely need to taper off slowly when I eventually quit. Overall negative effects are fairly minimal; I notice Kratom does tend to cause me to urinate more frequently (I usually consume it as tea which obviously adds to that issue), and on occasions this has been a real problem when drinking tea before bed. Taking Kratom (especially at higher doses) at night before bed definitely affects the quality of my sleep, so I’m trying to cut back/avoid doing that as much as possible. I do keep track of how much I take on a day-to-day basis to avoid increasing my average daily dose.”

Note: Aside from the adding quotation marks, open text responses from participants have been kept in their original form.

### 3.5 Dose-Related Associations With Pooled VAS Effects Ratings

Table 5 displays results from regression analyses using only data from respondents who reported currently being or having previously been regular kratom users (n = 104, 80.6%).

**TABLE 5:** Model 1 displays results from a multiple regression that examines pooled VAS ratings of beneficial effects from kratom use. Model 2 examines pooled VAS ratings of adverse or unwanted effects when kratom is not used for ≥1 day.

Model 1: Beneficial or positive effects from kratom use indications	B	95% CI	t	p	VIF
Intercept	76.95	[68.83, 87.1]	15.09	>0.01	
Age	−1.91	[−5.31, 1.49]	−1.11	0.27	1.04
Gender (male vs. female)	−0.35	[−7.44, 6.75]	−0.1	0.92	1.14
Race/Ethnicity (minority vs. white)	2.16	[−5.59, 9.90]	0.55	0.58	1.1
Education (high school vs. college graduate)	1.99	[−5.72, 9.70]	0.51	0.61	1.28
Employment (unemployed vs. employed)	−2.77	[−11.1, 5.51]	−0.66	0.51	1.3
Below US Federal poverty line for past−year annual income	4.34	[−4.86, 13.54]	0.93	0.35	1.32
Currently “regular” kratom user	−6.76	[−14.84, 1.32]	−1.66	0.1	1.32
Kratom dose consumed per week	2.19	[−1.52, 5.89]	1.17	0.25	1.19
Weeks of regular kratom use	0.9	[−2.98, 4.78]	0.46	0.65	1.11
Decreasing kratom dose (vs. unchanged dose)	0.27	[−9.39, 9.93]	0.06	0.96	1.66
Increased kratom dose (vs. unchanged dose)	−1.3	[−11.1, −8.52]	0.26	0.79	1.66
Quit kratom (vs. unchanged dose)	−16.45	[−25.9, −6.90]	3.42	0.01	1.66
Using kratom more outside of first waking hour	−2.65	[−1.00, 4.69]	−0.72	0.48	1.18
*F*(13,90) = 2.18, *p* = 0.02; R^2^ = 0.21; Adj R^2^ = 0.11					
Model 2: Adverse effects when kratom is not used for a period of >1 day					
Intercept	50.88	[35.87, 65.87]	6.78	>0.01	
Age	2.31	[−3.14, 7.76]	0.85	0.4	1.04
Gender (male vs. female)	−10.54	[−21.21, 0.14]	−1.97	0.05	1.14
Race/Ethnicity (minority vs. white)	−2.88	[−15.06, 9.30]	−0.47	0.64	1.1
Education (high school vs. college graduate)	−3.22	[−14.83, 8.40]	−0.55	0.58	1.28
Employment (unemployed vs. employed)	−2.63	[−16.21, 10.94]	−0.39	0.7	1.3
Below US Federal poverty line for past-year annual income	20.06	[5.21, 34.91]	2.7	0.01	1.32
Currently “regular” kratom user	−1.29	[−13.86, 11.28]	−0.2	0.84	1.32
Kratom dose consumed per week	5.24	[−0.52, 11.00]	1.82	0.07	1.19
Weeks of regular kratom use	6.74	[0.88, 12.60]	2.3	0.02	1.66
Decreasing kratom dose (vs. unchanged dose)	20.88	[5.75, 35.99]	2.76	0.01	1.66
Increased kratom dose (vs. unchanged dose)	11.4	[−2.18, 24.98]	1.68	0.1	1.66
Quit kratom vs. unchanged dose	−5.05	[22.48, 12.38]	−0.58	0.56	1.11
Using kratom more outside of first waking hour	6.1	[−5.75, 17.94]	−1.03	0.31	1.18
*F*(13,90) = 4.22, *p* < 0.001; R^2^ = 0.39; Adj R^2^ = 0.26					

Note: All models use responses from those who reported being, or previously having been, a regular kratom user (n = 104).

VIF, variance inflation factor.

The linear regression analyses of kratom dose and other related variables on pooled VAS ratings of beneficial kratom effects was significant [F(13,90) = 2.18, *p* = 0.02], with R2 = 0.21 and adjusted R2 = 0.11, however only having quit kratom entirely was significantly associated with lower positive ratings of beneficial motivating effects (*β* = −16.45, 95% CI = −25.9, −6.90; *p* < 0.001). Predictors that reflected dosing changes (e.g., self-reported amount of kratom consumed per week, weeks of regular use) were not significant.

The linear regression analyses of pooled VAS ratings of unwanted effects of ≥1 day cessation was also significant [F(13,90) = 4.22, *p* < 0.001], with R^2^ = 0.39 and adjusted R^2^ = 0.26. In this model, greater severity of unwanted effects was predicted by more weeks of regular kratom use (*β* = 6.74, 95% CI = 0.88, 12.60; *p* = 0.02), and having decreased kratom doses after initiation (*β* = 20.88, 95% CI = 5.75, 35.99; *p* < 0.001). The variable of greater self-reported amount of kratom consumed per week closely approached, but did not fully achieve, significance (*β* = 5.24, 95% CI = −0.52, 11.0; *p* = 0.07). Moreover, likelihood of experiencing negative effects from ≥1 day of kratom cessation was also higher in women (β = −10.54, 95% CI = −21.21, 0.14; *p* = 0.05) and for people reporting annual household income below the poverty line (β = 20.06, 95% CI = 5.21, 34.91; *p* < 0.001).

## 4 Discussion

We enrolled a diverse sample of US adults with reported lifetime kratom use to sensitively characterize patterns of kratom use and associated outcomes. These data add to our existing knowledge base through the inclusion of persons who have kratom-use histories but may not be currently consuming kratom: under half of the sample considered themselves to be current “regular” kratom users and 20.9% had quit kratom. The fact that most respondents had used kratom >100 times since initiation, over 80% had ever used kratom ≥4 times per week, and fewer than 10% had *never* taken kratom regularly indicates that the respondent sample had extensive and highly differentiated experience with kratom. Thus, the data represented here extend beyond prior studies that sampled persons with current kratom use to provide an updated perspective on kratom experiences.

### 4.1 Kratom Use as Another Routine of Daily Living or a Form of Drug Misuse?

Here noteworthy observations on kratom-use histories and patterns of (and changes in) use were observed. Consistent with prior research (Garcia-Romeu et al., 2020), persons in this study reported initiating kratom use at an older age and a minority had ever met criteria for KUD (Smith et al., 2022). This study also administered measures that have been associated with dependence severity for other substances in a modified format in order to assess dependence severity for kratom in our sample. The results suggest that a subset of respondents endorsed several behaviors indicative of greater dependence severity. Yet the nature of kratom use seems different from other substances and makes the interpretation of these data challenging. For instance, we observed that 60% of our respondents had used kratom >100 times during their lifetime. This value is widely accepted as evidence of someone being a verified “cigarette smoker” and would probably raise clinical concerns in reference to a drug such as heroin or cocaine. However, such an exposure would generally be considered normative for drugs such as alcohol, caffeine, or most psychiatric medications (such as SSRIs). Functionally and socially, these classes of drugs are distinguished from each other in terms of a major component of DSM criteria for SUDs: the extent to which their acute and chronic effects tend to be concordant with the goals and obligations of everyday life. We asked our respondents to characterize kratom in exactly that way: first, whether it had acute effects with each dose (like caffeine or alcohol, but unlike most psychiatric medications), and second, whether such acute effects were compatible with daily goals and obligations (perhaps like the effects of caffeine, and probably not like the effects of alcohol). Only 7% of our respondents stated categorically that they never felt acute effects from kratom, and—even with the broadest interpretation of the free-text responses—it appears that no more than 9% of respondents (n = 12) used kratom for chronic effects only (the way they might have used a psychiatric medication or other maintenance medication). Thus, for most respondents, the clinically and functionally relevant question about kratom is whether its acute effects were compatible with their daily goals and obligations.

The answer to that question was usually yes (for 86/120 respondents who ever experienced acute effects: 70%), with only three reporting that the acute effects outright *undermined* their daily goals or obligations (and nine more saying that they did not take enough kratom to judge). For those 86 respondents, a history of >100 exposures to kratom might be functionally comparable to a history of >100 exposures to caffeine—though we hasten to add that the comparison is not a straightforward one. Apart from the obvious fact that the two substances represent different pharmacological classes (with kratom having opioid activity), there is a host of unknowns specific to kratom. Kratom is a relatively recent introduction to US markets, and its complex and variable pharmacology are only beginning to be understood. That understanding is hampered further by a lack of standardization of kratom products. We suggest caffeine as a point of comparison in only the following functional ways: both substances are available without a prescription in a variety of dosage forms; both usually produce acute effects with each dose (despite also producing substantial tolerance), and the acute effects are usually described as helpful toward meeting daily goals or obligations; both may lead to withdrawal symptoms on cessation (Juliano and Griffiths, 2004; Bowe and Kerr, 2020; Stanciu et al., 2021); and both are occasionally used to excess, with adverse effects (Reissig et al., 2009; Schmuhl, et al., 2020), though the use of either substance for purely euphoriant purposes is more the exception than the rule (McCarthy et al., 2008; Smith et al., 2021b).

The similarities may end there, both for worse and for better. Kratom seems to have potential for instrumental “self-treatment” of chronic pain, a variety of psychological or psychiatric symptoms, and SUDs (Smith et al., 2021b; Smith et al., 2021c). For most respondents, during periods of regular kratom use, the frequency of changes in dosing routine was either “not often” or “never,” suggesting arrival at a pattern of use helpful for daily functioning. Even so, some of our respondents did *increase* their dosages, and a few specified that they were continuing to use kratom despite tolerance because they hoped once again to feel acute effects. Other respondents had *decreased* their dosages, or quit, citing unwanted effects. Variability in dose-effect relationships is further underscored by our respondents’ varied patterns of dosage timing: the first dose of the day occurred within an hour of waking for about 45% of our respondents, but later in the day for the other 55%. This suggests that *proximal* motivations for use of kratom need to be assessed and understood at the individual and momentary level, because there may be considerable differences in whether the effects are perceived (and under what contexts) as mostly energizing, mostly calming, or some combination of the two.

### 4.2 Effects and Effectiveness

Among the most intriguing findings here is how close average doses were in terms of being reported as ineffective, effective, or “a bit too much.” The ranges were similar for all those ratings of effectiveness based on prior findings (Garcia-Romeu et al., 2020; Smith et al., 2021b). The highest average dose for any dose type that was considered “a bit too much” was 8.68 g, compared to 6.85 g, which was reported as effective. This was followed by 7.25 capsules (“too much”) versus 5.88 capsules (effective); 3.93 spoonfuls (“too much”) versus 2.87 (effective); and 3.44 cups of tea (“too much”) versus 2.25 (effective). These ranges are in keeping with the typical regular doses reported in other surveys. There are several takeaways from this, the first being that most in this sample were not typically using extremely high doses of kratom. The other takeaway is that the average difference between effective kratom doses and doses that were perceived as “too much” (and unwanted) is not large, meaning that people using kratom, particularly those unfamiliar with kratom, may inadvertently dose too much. Strong conclusions cannot be made, in part, due to the variability of kratom products and batches of product likely used among the sample. For example, most capsules appear to contain about 0.5 g among what is primarily being sold for kratom powders. This would roughly translate as the powder being about half as much as the number of capsules. However, this may not always be true if larger capsules are used. Presently capsules not self-prepared by those who consume kratom can be purchased in “regular” or “jumbo” sized.

Overall, the pooled VAS ratings for therapeutic or beneficial effects of kratom were higher on average than were severity ratings for withdrawal-like effects upon 1 day’s cessation, among people who had ever regularly used kratom (including among people who had quit kratom but whom had once used regularly). These findings again provide a complicated picture insofar as there is reported benefit, but clearly also adverse effects, both when kratom is used (as indicated in open text responses) and when use is paused for at least a day. Many of the cessation symptoms, along with the direct adverse effects described, were similar to what would be expected from opioids.

We were unable to find strong person-level predictors of proneness to beneficial effects, withdrawal-like effects, or adverse effects. Our regression models showed mostly that respondents who did not note a preponderance of benefits were those who had quit. Amount of use per week and duration of use were not associated in either direction with beneficial effects, though they were associated with higher severity of withdrawal-like effects. As our goal was to detect any signal of a dose-effects relationship, these findings should be taken as exploratory and as a starting point for refining methods. They do generally comport with prior findings from the US and Asia that kratom withdrawal may be dependent on both dose and duration of use, but is typically mild to moderate, and severe among a minority (Ahmad and Aziz, 2012; Singh et al., 2014; Singh et al., 2018; Singh et al., 2019b; Stanciu et al., 2019; Garcia-Romeu et al., 2020; Smith et al., 2021b). Ambiguity is attributable in part to the sample size, but also again a likely artifact of the variability of kratom product types. Extracts are far more potent in terms of their alkaloid concentration for MG and 7-HG (and typically more expensive) but may have greater alkaloid purity. Conversely, pulverized plant matter may be less potent, but also may be of poorer quality or, if purchased from a less reputable vendor who does not comport to the Good Manufacturing Standards Program guidelines, may be adulterated. Moreover, it is important to underscore that this is a US sample, meaning that it is unlikely that any respondents had access to fresh, raw kratom leaves.

Ultimately, findings here support the possibility that regular kratom dosing and longer duration of regular use in weeks is associated with higher ratings of adverse effects when kratom is not used for a day or more, but that they are mild to moderate. Although we did not detect clear relationships between kratom dose and direct beneficial effects from use, that could be due to methodological and statistical power limitations. However, that pooled VAS ratings were higher for beneficial effects from use than for ratings for adverse effects from 1 day of cessation does partially support the narrative that has emerged from prior self-report among current kratom-using adults, namely that many who have regularly used kratom tend to experience beneficial effects but without the seeming severity of adverse outcomes associated with illicit psychoactive drugs.

Like all findings, these constitute provisional takeaways. The patterns of and changes in dosing here clearly show the variability of kratom use experiences. Perhaps the most important finding is among the simplest: not everyone who has used kratom regularly or irregularly continues to use it. The reasons for continued regular kratom use, primarily for self-treating pain, psychiatric, or SUD symptoms are increasingly established (Grundmann, 2017; Coe et al., 2019; Bath et al., 2020; Garcia-Romeu et al., 2020). The reasons for *ir*regular or discontinued use remain less clear.

## 5 Limitations

One of the main limitations of this study is the cross-sectional design of the survey and the small sample size. The use of a large crowdsourcing platform is a strength in some ways (such as wide geographical coverage in a short time), but also means that findings are not generalizable to all people with kratom use histories, including those who engage with other platforms. Similar to large US online surveys that recruited current kratom-using adults, there is the possibility that those who choose to participate in this kratom survey had particular attitudes toward kratom. As we were able to recruit persons who still used kratom regularly as well as those who had stopped use, the sample is diverse, but may still reflect different biases regarding kratom. Among those who had stopped using kratom, or who had used it only ever intermittently, recall bias may be a concern. Additionally, the small sample size did not permit for precise estimates. Likewise, the kratom products that people reported dosing must be presumed to reflect products varied in both alkaloid content and quality; and we cannot know if any were adulterated. Concomitant use of kratom with other substances and dietary habits that could potentiate or attenuate kratom effects may also have occurred, thus limiting conclusions that might be drawn, including kratom’s beneficial or adverse effects and effectiveness when used alone. Lastly, it is critical to keep in mind that heterogeneity of kratom products within the US is considerable, but also that commercialized and processed kratom products consumed in the US likely differ from fresh kratom preparations available and used in Southeast Asia (Saref et al., 2019; Charoenratana et al., 2021; Kamble et al., 2021).

## 6 Conclusion

The regular use of kratom in the US continues to grow at unknown rates, making it incumbent upon researchers and healthcare providers to include kratom use history items on broader surveys related to substance use or clinical assessments. Here, by using a survey sample not selected in advance for current kratom use, we uncovered an important issue of potential survivor bias in currently available data. Our findings, while preliminary, also show the diversity among kratom users in terms of dosing methods. Although a strong relationship between dose and pooled effects could not be found for beneficial effects directly associated with kratom use, and with limited precision for adverse effect ratings, it is likely that such relationships can be achieved through improved survey methods and increased sample size. Still, cross-sectional methods will be insufficient for attaining a scientific understanding of kratom dose-effect relationships, particularly when samples of kratom product types used by respondents are not also assessed. There is no peer-reviewed research about safe or effective dosing of kratom, and this paucity of information plays out in variability in doses taken (resulting in either potential increased risk or subtherapuetic dose). The variability in kratom leaves and products means that dose-effect relationships in everyday settings may be difficult to discern without also pairing self-report with assay of the kratom product being used. Although we intend to improve such methods, we anticipate similar limitations in assessment without objective data. One take-away from this exploratory characterization is that controlled human laboratory studies are critical to advancing this area further. Direct observation and assessment using validated measures of both subjective and objective acute effects and withdrawal are needed.

In the interim, we can say that most persons who use kratom experience at least some acute effects with every dose, and that those acute effects are usually seen as compatible with, or even helpful for, daily obligations (i.e., kratom is seemingly not typically used like prescribed medications that confer perceived quality-of-life benefits only chronically, but perhaps perceived more similiarly to coffee or even alcohol). We found indications that higher weekly kratom dose amounts and longer periods of regular use were associated with greater severity ratings for unwanted effects when kratom was not used for at least a day. The clinical relevance is that persons who use kratom at higher doses regularly may expect greater odds of feeling unwanted or adverse effects when use is paused. Ultimately, that pooled ratings of beneficial effects from kratom use were higher than pooled ratings of adverse effects from discontinuation for at least a day, present us with yet another layer of complexity in understanding the *perceived* benefits of kratom use versus harm or risk. The *actual* therapeutic benefits and risks of kratom, which will necessarily include subjective assessments of effectiveness or ineffectiveness of kratom at various doses among human subjects, remains to be elucidated. The public health message regarding kratom thus remains incomplete and will likely remain so until controlled human laboratory experiments are underway. Presently, persons who use kratom, and clinicians who encounter kratom-using patients, should remain cognizant of dosing regimens and dosing changes and work to document observed effects. The margin between effective doses and doses that were perceived as “too much” appears narrow enough to warrant careful attention to kratom doses, irrespective of a given dosing unit.

## Data Availability

The raw data supporting the conclusion of this article will be made available by the authors, without undue reservation.
