# Supplementary material for: Searching for a Signal: Self-Reported Kratom Dose-Effect Relationships Among a Sample of US Adults With Regular Kratom Use Histories
Source: Front Pharmacol. 2022 Mar 1;13:765917. doi: 10.3389/fphar.2022.765917 (PMC8921773; doi:10.3389/fphar.2022.765917)

Supplementary Material

# Supplementary Tables

## Supplementary Table 1. Survey items and response options that participants could select. All responses were rated by participants using visual analogues scales (0-100) for severity of effect^a^.

| *“Please select from the below list the most important factors that influence or motivate your current kratom use (if you still use) or past kratom use (if you no longer use). In other words, what are your most important reasons for your using kratom during times when you used it? Select all that apply.”* | *“What unpleasant or unwanted side effects have you experienced when you have stopped taking kratom at least for a period of one day or longer? These could be considered withdrawal or withdrawal-like effects?”* |
| --- | --- |
| Self-treat anxiety symptoms | Anxiety |
| Relieve short-term pain (acute pain management). | Irritability |
| Reduce social anxiety. | Low energy |
| Self-treat depression symptoms. | Desire to use kratom |
| Self-treat long-term pain issues and symptoms (chronic pain management). | Restlessness/unable to sit still |
| Self-treat headaches/migraines. | Trouble sleeping |
| Self-treat chronic fatigue syndrome. | Nausea |
| Self-treat ADD/ADHD symptoms. | Body aches or pains |
| Self-treat post-traumatic stress symptoms. | No energy/lethargic |
| Self-treat irritable bowel syndrome. | Upset stomach (aches, diarrhea, etc.) |
| Self-treat bipolar symptoms. | Depressed or sad |
| Relieve withdrawal symptoms from nonprescribed opioids or heroin. | Mild-moderate craving for kratom |
| Relieve withdrawal symptoms from medically prescribed opioids. | Sleepiness/fatigue |
| Relieve withdrawal symptoms from a variety of different drugs. | Hot flashes |
| Relieve alcohol withdrawal symptoms. | Running nose |
| Relieve withdrawal symptoms from nonprescribed buprenorphine (Suboxone, Subutex). | Restless legs |
| Relieve withdrawal symptoms from buprenorphine (Suboxone, Subutex). | Craving for another substance to help relieve kratom withdrawal symptoms |
| Relieve withdrawal symptoms from nonprescribed methadone. | Cold flashes |
| Relieve withdrawal symptoms from “nootropics”/ “noots”, “cognitive enhancing” supplements. | Watery eyes |
| Relieve withdrawal symptoms from prescribed methadone. | Vomiting |
| As a short-term substitute/replacement for opioids (e.g., prescription opioids, heroin). | Intense craving for kratom |
| Couldn’t get ahold of other, more preferred drugs. |  |
| A short-term substitute/replacement for alcohol. |  |
| Doctors won’t prescribe the drugs you need. |  |
| As a long-term substitute/replacement for opioids (e.g., prescription opioids, heroin). |  |
| A short-term substitute/replacement for stimulants (meth, cocaine). |  |
| A long-term substitute/replacement for alcohol. |  |
| A long-term replacement for buprenorphine (Suboxone, Subutex). |  |
| A long-term substitute/replacement for stimulants (meth, cocaine). |  |
| A short-term replacement for buprenorphine (Suboxone, Subutex). |  |
| A short-term replacement for methadone. |  |
| A long-term replacement for prescribed methadone. |  |
| Difficulties obtaining buprenorphine (Suboxone, Subutex) or methadone. |  |
| Just to feel less crappy in general and improve your quality of life. |  |
| Address occasional feelings of sleepiness or low energy. |  |
| For recreation, fun, or to relax. |  |
| Boost energy, stamina and/or endurance (for work, exercise). |  |
| Kratom is safer than other substances. |  |
| To achieve a euphoric high. |  |
| To take as part of a self-designed “stack” of other drugs that help you feel good. |  |
| Because you prefer the kratom “high” to “highs” you get from other drugs. |  |

**^a^** Proportions and means from these items will be reported elsewhere. They were pooled and averaged and reported in that form only for the purposes of analyses conducted for this study.

## Supplementary Table 2.

| **Pearson's Correlations** | | | | | | | | |
| --- | --- | --- | --- | --- | --- | --- | --- | --- |
| **Variable** |  | **Kratom amount / week** | **Age** | **Kratom Initiation Age** | **Weeks of using regular dose** | **Pooled Positive VAS** | **Pooled Withdraw VAS** | **Weeks of any kratom use** |
| **Kratom amount / week** | Pearson's r | — |  |  |  |  |  |  |
|  | p-value | — |  |  |  |  |  |  |
| **Age** | Pearson's r | 0.000 | — |  |  |  |  |  |
|  | p-value | 0.992 | — |  |  |  |  |  |
| **Kratom Initiation Age** | Pearson's r | -0.067 | *0.913* | — |  |  |  |  |
|  | p-value | 0.498 | *< .001* | — |  |  |  |  |
| **Weeks of using regular dose** | Pearson's r | 0.144 | 0.135 | -0.027 | — |  |  |  |
|  | p-value | 0.146 | 0.173 | 0.789 | — |  |  |  |
| **Pooled Positive VAS** | Pearson's r | 0.081 | -0.037 | -0.013 | 0.138 | — |  |  |
|  | p-value | 0.411 | 0.707 | 0.893 | 0.162 | — |  |  |
| **Pooled Withdraw VAS** | Pearson's r | 0.036 | 0.155 | 0.055 | *0.326* | 0.119 | — |  |
|  | p-value | 0.714 | 0.116 | 0.582 | *< .001* | 0.228 | — |  |
| **Weeks of any kratom use** | Pearson's r | 0.142 | 0.115 | -0.034 | *0.865* | 0.173 | *0.338* | — |
|  | p-value | 0.151 | 0.246 | 0.73 | *< .001* | 0.079 | *< .001* | — |

## Supplementary Table 3.

| **Student's t-test (Grouping Variables ~ Amount of Kratom Consumed Per Week)** | | | | | | | |  |
| --- | --- | --- | --- | --- | --- | --- | --- | --- |
| **Group** | **N** | **Mean** | **SD** | **SE** | **t** | **df** | **p** | **Cohen's d** |
| Female | 54 | -0.074 | 0.907 | 0.123 | -1.983 | 102 | 0.05 | -0.389 |
| Male | 50 | 0.329 | 1.162 | 0.164 |  |  |  |  |
|  |  |  |  |  |  |  |  |  |
| Minority | 28 | -0.05 | 0.719 | 0.136 | -0.997 | 102 | 0.321 | -0.22 |
| White | 76 | 0.182 | 1.149 | 0.132 |  |  |  |  |
|  |  |  |  |  |  |  |  |  |
| College | 63 | -0.084 | 0.739 | 0.093 | -2.508 | 102 | *0.014* | -0.503 |
| Highschool | 41 | 0.433 | 1.356 | 0.212 |  |  |  |  |
|  |  |  |  |  |  |  |  |  |
| Employed | 72 | 0.152 | 1.131 | 0.133 | 0.464 | 102 | 0.644 | 0.098 |
| Unemployed | 32 | 0.048 | 0.86 | 0.152 |  |  |  |  |
|  |  |  |  |  |  |  |  |  |
| Above Poverty Line Annual Income | 82 | 0.206 | 1.149 | 0.127 | 1.623 | 102 | 0.108 | 0.39 |
| Below Poverty Line Annual Income | 22 | -0.201 | 0.449 | 0.096 |  |  |  |  |
|  |  |  |  |  |  |  |  |  |
| Currently Considers Themselves a Regular Kratom User | 53 | 0.287 | 1.137 | 0.156 | 1.664 | 102 | 0.099 | 0.326 |
| Not currently a regular kratom user | 51 | -0.054 | 0.936 | 0.131 |  |  |  |  |
|  |  |  |  |  |  | | | |
| More Within 1st Waking Hour | 63 | 0.25 | 1.078 | 0.136 | 1.577 | 102 | 0.118 | 0.316 |
| Less Within 1st Waking Hour | 41 | -0.081 | 0.991 | 0.155 |  |  |  |  |

## Supplementary Table 4.

| **ANOVA (Grouping Variables ~ Amount of Kratom Consumed Per Week)** | | | | | |  |
| --- | --- | --- | --- | --- | --- | --- |
| **Cases** | **Sum of Sq.** | **df** | **Mean Sq.** | **F** | **p** | **η²** |
| Is kratom compatible with daily obligations? | 0.731 | 2 | 0.366 | 0.326 | 0.723 | 0.006 |
| Residuals | 113.318 | 101 | 1.122 |  |  |  |
| **Daily Obligations** | **Mean** | **SD** | **N** |  |  |  |
| No | 0.309 | 1.029 | 17 |  |  |  |
| Yes, helpful | 0.087 | 1.059 | 62 |  |  |  |
| Yes, not helpful | 0.071 | 1.08 | 25 |  |  |  |
|  |  |  |  |  |  |  |
| **Cases** | **Sum of Sq.** | **df** | **Mean Sq.** | **F** | **p** | **η²** |
| Have you changed your kratom dosing since you started taking kratom? | 6.213 | 3 | 2.071 | 1.921 | 0.131 | 0.054 |
| Residuals | 107.837 | 100 | 1.078 |  |  |  |
| **Changed Dose?** | **Mean** | **SD** | **N** |  |  |  |
| Yes, decreased | -0.292 | 0.534 | 23 |  |  |  |
| Yes, increased | 0.382 | 1.116 | 33 |  |  |  |
| Quit | 0.172 | 1.449 | 18 |  |  |  |
| No, unchanged | 0.116 | 0.947 | 30 |  |  |  |

## Supplementary Table 5.

| **Student's t-test (Grouping Variables ~ Pooled Positive Effects)** | | | | |  |  |  |  |
| --- | --- | --- | --- | --- | --- | --- | --- | --- |
| **Group** | **N** | **Mean** | **SD** | **SE** | **t** | **df** | **p** | **Cohen's d** |
| Female | 54 | 71.887 | 20.66 | 2.811 | -0.601 | 102 | 0.549 | -0.118 |
| Male | 50 | 73.867 | 11.174 | 1.58 |  |  |  |  |
|  |  |  |  |  |  |  |  |  |
| Minority | 28 | 70.388 | 19.368 | 3.66 | -0.906 | 102 | 0.367 | -0.2 |
| White | 76 | 73.742 | 15.698 | 1.801 |  | | | |
|  |  |  |  |  |  |  |  |  |
| College | 63 | 71.373 | 18.594 | 2.343 | -1.108 | 102 | 0.27 | -0.222 |
| Highschool | 41 | 75.091 | 13.287 | 2.075 |  |  |  |  |
|  |  |  |  |  |  |  |  |  |
| Employed | 72 | 72.086 | 16.143 | 1.902 | -0.687 | 102 | 0.494 | -0.146 |
| Unemployed | 32 | 74.534 | 18.145 | 3.208 |  |  |  |  |
|  |  |  |  |  |  |  |  |  |
| Above Poverty Line Annual Income | 82 | 72.681 | 17.229 | 1.903 | -0.184 | 102 | 0.854 | -0.044 |
| Below Poverty Line Annual Income | 22 | 73.426 | 15.102 | 3.22 |  |  |  |  |
|  |  |  |  |  |  |  |  |  |
| Currently Considers Themselves a Regular Kratom User | 53 | 76.381 | 14.254 | 1.958 | 2.243 | 102 | *0.027* | 0.44 |
| Not currently a regular kratom user | 51 | 69.158 | 18.396 | 2.576 |  |  |  |  |
|  |  |  |  |  |  |  |  |  |
| More Within 1st Waking Hour | 48 | 72.339 | 14.793 | 2.135 | -0.281 | 102 | 0.78 | -0.055 |
| Less Within 1st Waking Hour | 56 | 73.267 | 18.357 | 2.453 |  |  |  |  |

## Supplementary Table 6.

| **ANOVA (Grouping Variables ~ Pooled Positive Effects)** | | | | | | |
| --- | --- | --- | --- | --- | --- | --- |
| **Cases** | **Sum of Sq.** | **df** | **Mean Sq.** | **F** | **p** | **η²** |
| Is kratom compatible with daily obligations? | 7169.896 | 2 | 3584.948 | 16.708 | *< .001* | 0.249 |
| Residuals | 21671.592 | 101 | 214.57 |  |  |  |
| **Daily Obligations** | **Mean** | **SD** | **N** |  |  |  |
| No | 54.833 | 24.508 | 17 |  |  |  |
| Yes, helpful | 77.999 | 12.96 | 62 |  |  |  |
| Yes, not helpful | 72.285 | 8.696 | 25 |  |  |  |
|  |  |  |  |  |  |  |
| **Cases** | **Sum of Sq.** | **df** | **Mean Sq.** | **F** | **p** | **η²** |
| Have you changed your kratom dosing since you started taking kratom? | 2001.936 | 3 | 667.312 | 2.486 | 0.065 | 0.069 |
| Residuals | 26839.552 | 100 | 268.396 |  |  |  |
| **Changed Dose?** | **Mean** | **SD** | **N** |  |  |  |
| Yes, decreased | 73.356 | 11.922 | 23 |  |  |  |
| Yes, increased | 75.593 | 13.304 | 33 |  |  |  |
| Quit | 63.42 | 22.654 | 18 |  |  |  |
| No, unchanged | 75.064 | 17.932 | 30 |  |  |  |

## Supplementary Table 7.

| **Student's t-test (Grouping Variables ~ Negative Effects)** | | | | |  |  |  |  |
| --- | --- | --- | --- | --- | --- | --- | --- | --- |
| **Group** | **N** | **Mean** | **SD** | **SE** | **t** | **df** | **p** | **Cohen's d** |
| Female | 54 | 55.67 | 22.80 | 3.10 | 1.36 | 102.00 | 0.18 | 0.27 |
| Male | 50 | 49.28 | 25.11 | 3.55 |  |  |  |  |
|  |  |  |  |  |  |  |  |  |
| Minority | 28 | 52.61 | 25.35 | 4.79 | 0.00 | 102.00 | 1.00 | 0.00 |
| White | 76 | 52.59 | 23.71 | 2.72 |  |  |  |  |
|  |  |  |  |  |  |  |  |  |
| College | 63 | 53.24 | 22.17 | 2.79 | 0.33 | 102.00 | 0.74 | 0.07 |
| Highschool | 41 | 51.62 | 26.92 | 4.20 |  |  |  |  |
|  |  |  |  |  |  |  |  |  |
| Employed | 72 | 55.50 | 22.21 | 2.62 | 1.87 | 102.00 | 0.06 | 0.40 |
| Unemployed | 32 | 46.06 | 26.95 | 4.76 |  |  |  |  |
|  |  |  |  |  |  |  |  |  |
| Above Poverty Line Annual Income | 82 | 52.63 | 24.16 | 2.67 | 0.03 | 102.00 | 0.98 | 0.01 |
| Below Poverty Line Annual Income | 22 | 52.48 | 24.13 | 5.15 |  |  |  |  |
|  |  |  |  |  |  |  |  |  |
| Currently Considers Themselves a Regular Kratom User | 53 | 56.94 | 23.72 | 3.26 | 1.90 | 102.00 | 0.06 | 0.37 |
| Not currently a regular kratom user | 51 | 48.09 | 23.76 | 3.33 |  |  |  |  |
|  |  |  |  |  |  |  |  |  |
| More Within 1st Waking Hour | 48 | 52.51 | 24.93 | 3.60 | -0.03 | 102.00 | 0.97 | -0.01 |
| Less Within 1st Waking Hour | 56 | 52.67 | 23.47 | 3.14 |  |  |  |  |

## Supplementary Table 8.

| **ANOVA (Grouping Variables ~ Pooled Negative Effects)** | | | | | | |
| --- | --- | --- | --- | --- | --- | --- |
| **Cases** | **Sum of Sq.** | **df** | **Mean Sq.** | **F** | **p** | **η²** |
| Is kratom compatible with daily obligations? | 333.281 | 2 | 166.64 | 0.284 | 0.753 | 0.006 |
| Residuals | 59171.078 | 101 | 585.852 |  |  |  |
| **Daily Obligations** | **Mean** | **SD** | **N** |  |  |  |
| No | 56.368 | 23.771 | 17 |  |  |  |
| Yes, helpful | 52.315 | 23.384 | 62 |  |  |  |
| Yes, not helpful | 50.736 | 26.438 | 25 |  |  |  |
|  |  |  |  |  |  |  |
| **Cases** | **Sum of Sq.** | **df** | **Mean Sq.** | **F** | **p** | **η²** |
| Have you changed your kratom dosing since you started taking kratom? | 4494.936 | 3 | 1498.312 | 2.724 | *0.048* | 0.076 |
| Residuals | 55009.423 | 100 | 550.094 |  |  |  |
| **Changed Dose?** | **Mean** | **SD** | **N** |  |  |  |
| Yes, decreased | 54.442 | 21.78 | 23 |  |  |  |
| Yes, increased | 60.818 | 21.015 | 33 |  |  |  |
| Quit | 48.246 | 28.923 | 18 |  |  |  |
| No, unchanged | 44.753 | 23.649 | 30 |  |  |  |

# Supplementary Figures

## Box and whisker plot displaying ranges of people’s self-reported kratom doses in grams of kratom, ranging from which dose they perceived to “ineffective” to what dose they perceived to be “too much”.

##
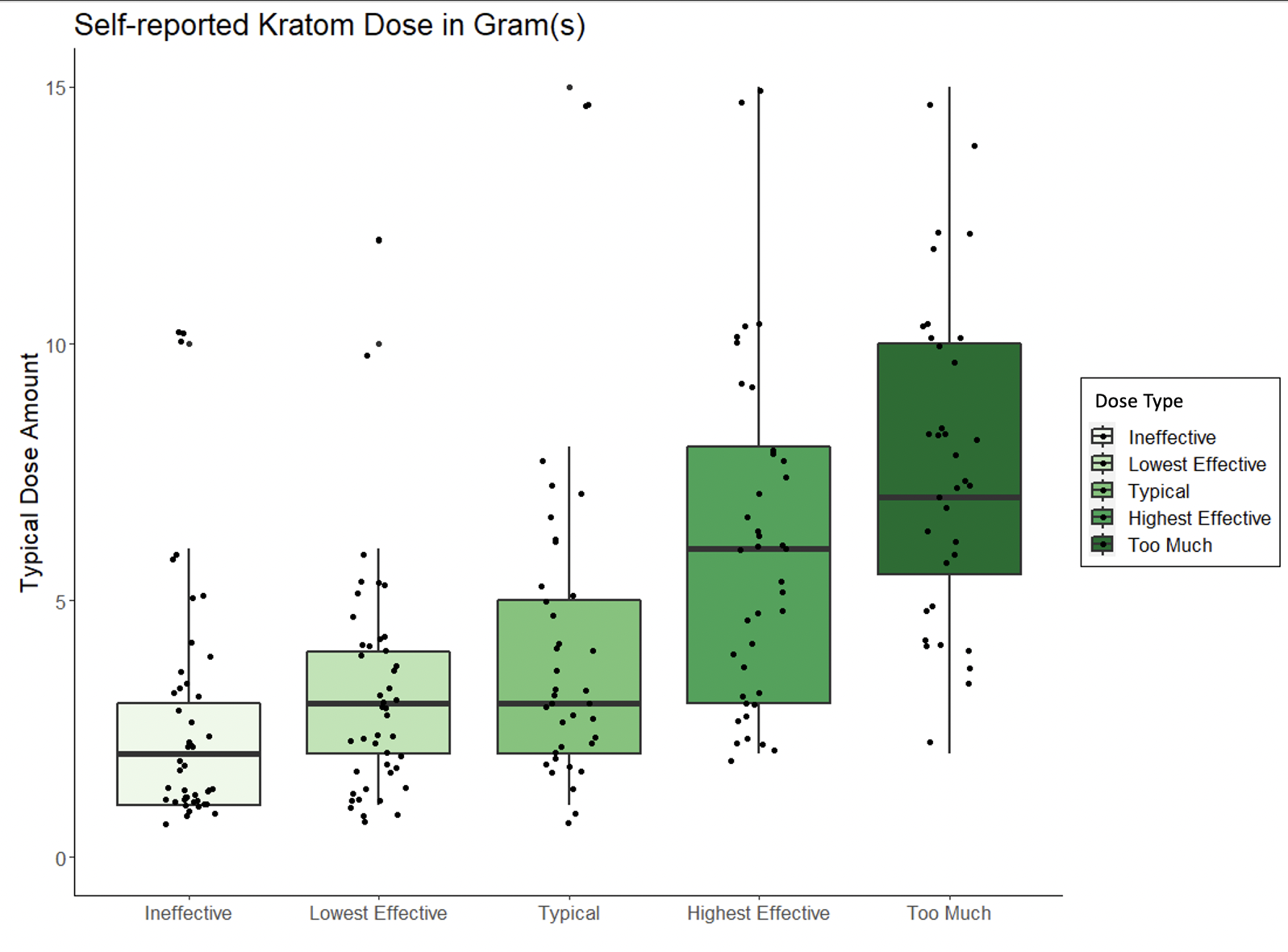


## Box and whisker plot displaying ranges of people’s self-reported kratom doses in capsules of kratom, ranging from which dose they perceived to “ineffective” to what dose they perceived to be “too much”.


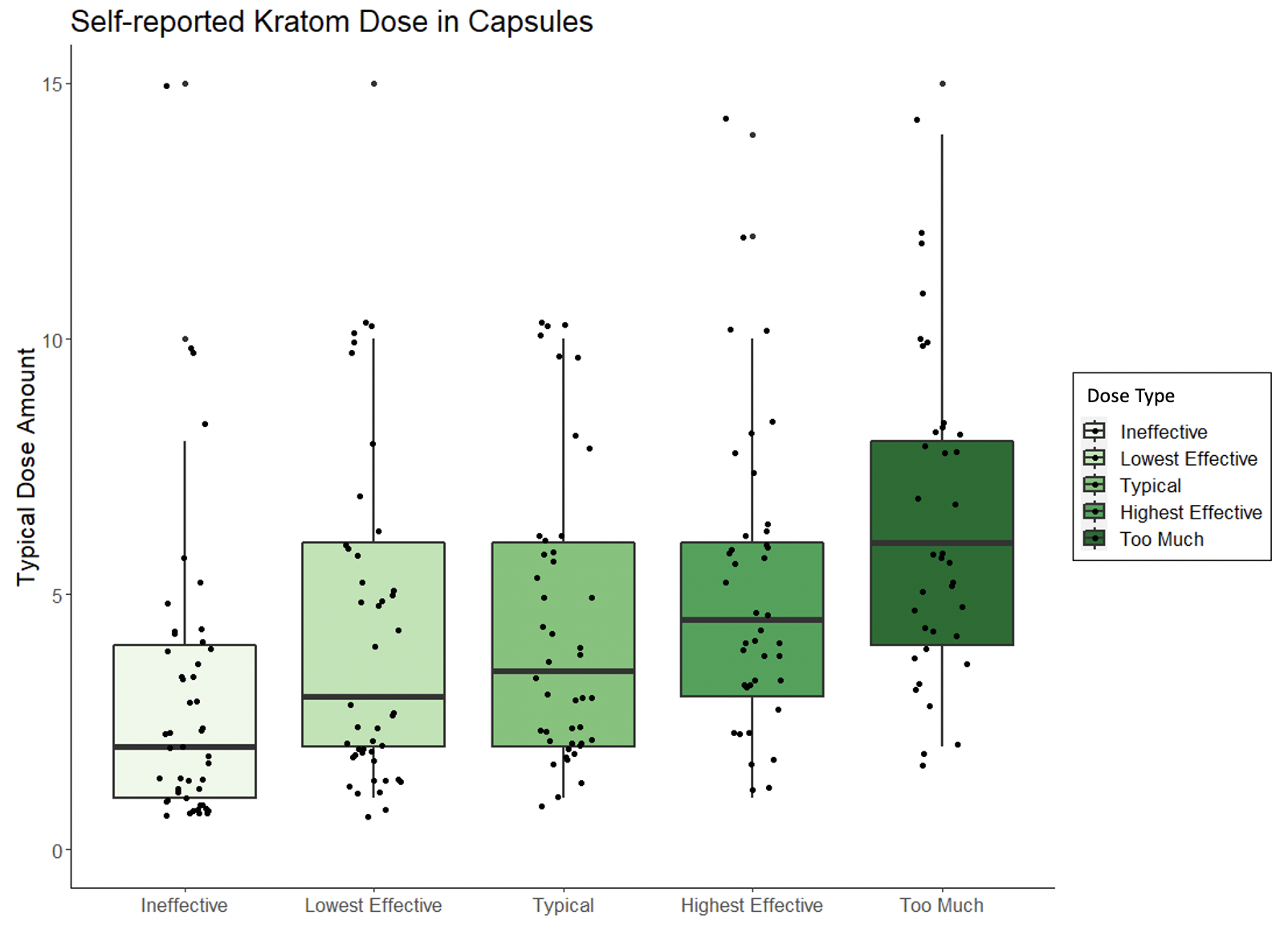


## Box and whisker plot displaying ranges of people’s self-reported kratom doses in spoonfuls of kratom, ranging from which dose they perceived to “ineffective” to what dose they perceived to be “too much”.


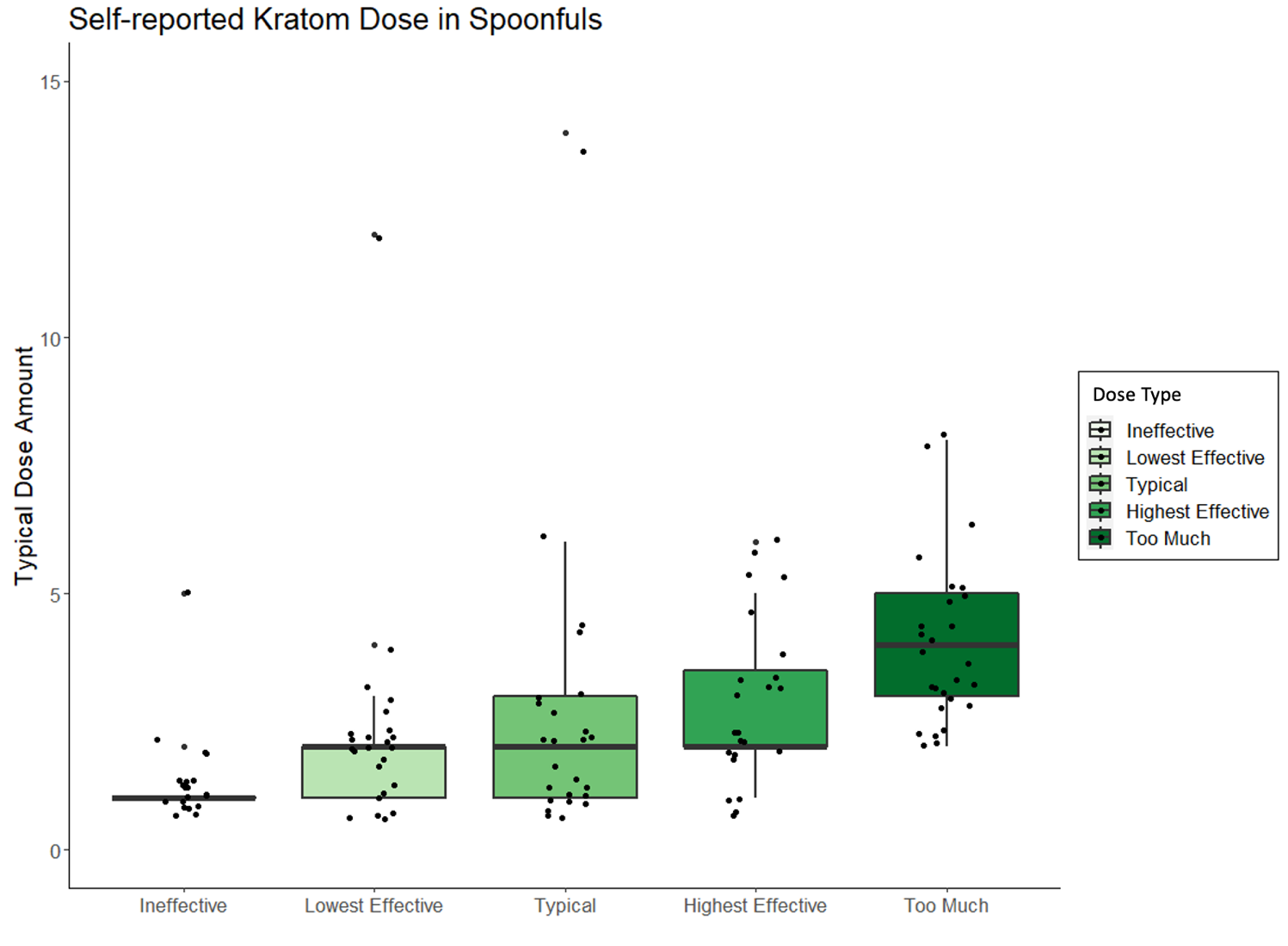


## Box and whisker plot displaying ranges of people’s self-reported kratom doses in tablespoons of kratom, ranging from which dose they perceived to “ineffective” to what dose they perceived to be “too much”.


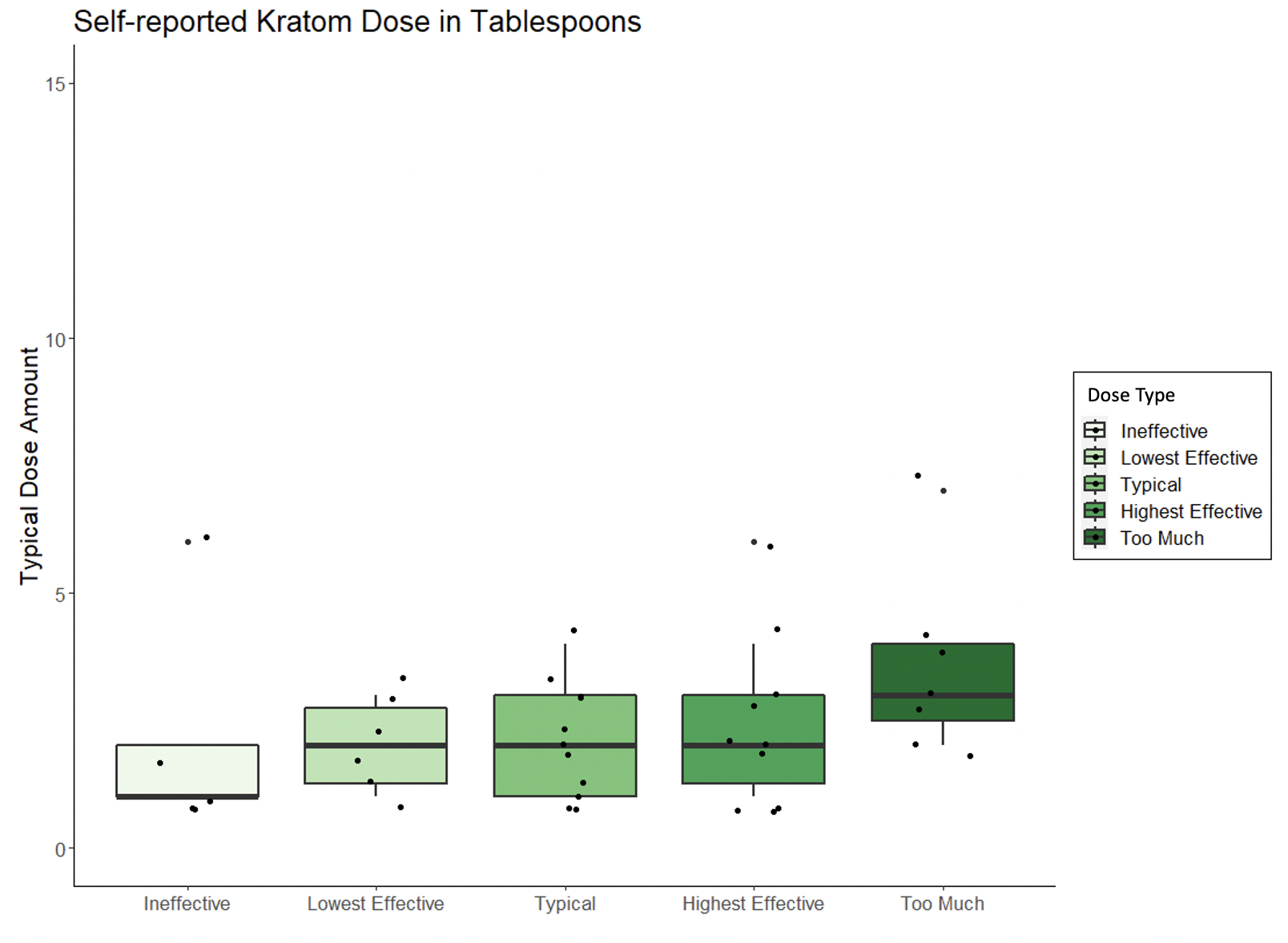


## Box and whisker plot displaying ranges of people’s self-reported kratom doses in cups of tea of kratom, ranging from which dose they perceived to “ineffective” to what dose they perceived to be “too much”.


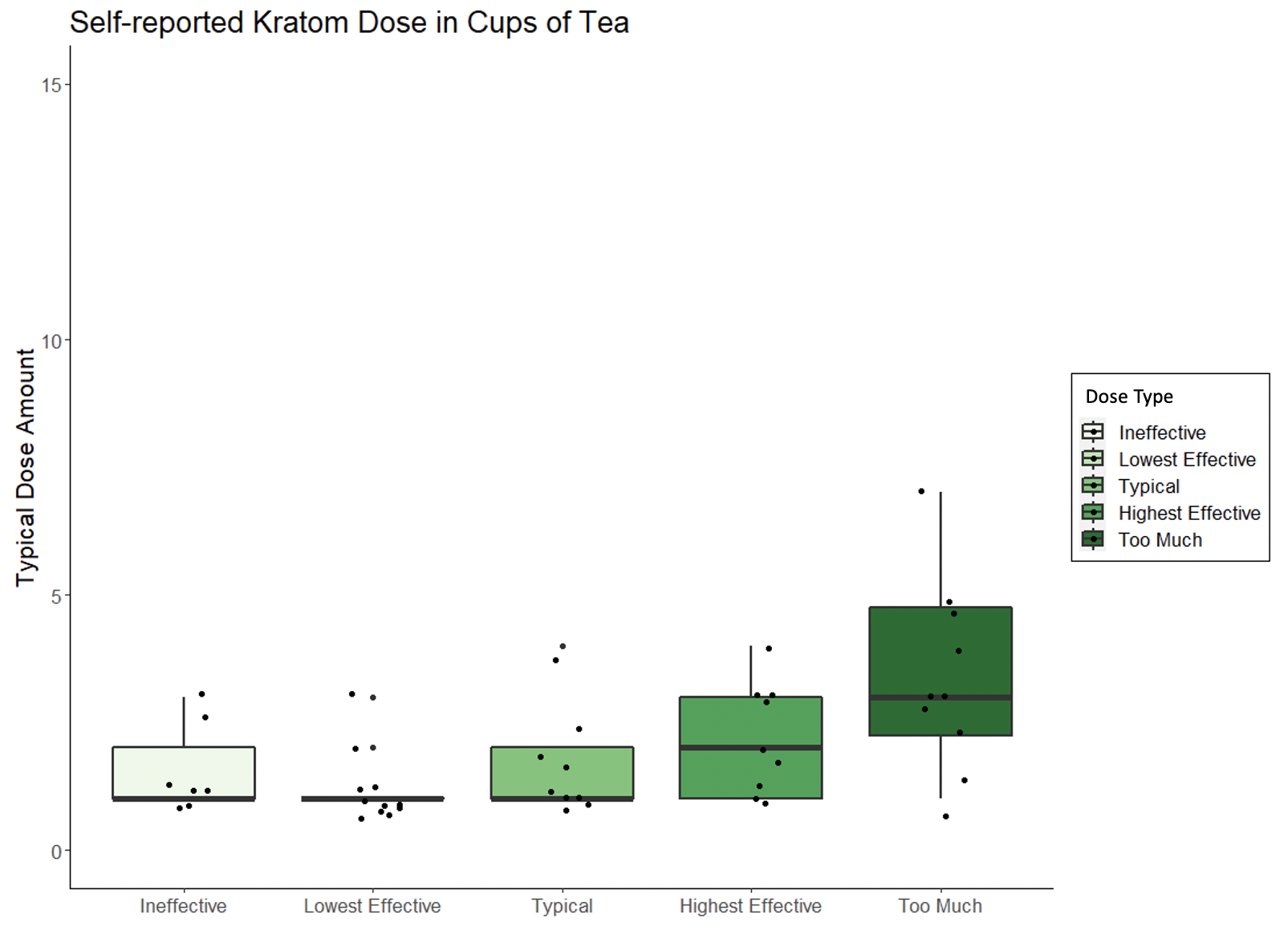

Supplement: Supplementary file 1 [file DataSheet1.docx]
